# Panel dataset on de jure central bank independence in 21 OECD countries (excluding the Eurozone)

**DOI:** 10.1016/j.dib.2024.110094

**Published:** 2024-02-01

**Authors:** Tomáš Pokorný, Adam Ruschka, Helena Chytilová

**Affiliations:** Prague University of Economics and Business, Czechia

**Keywords:** Central bank, OECD countries, Independence

## Abstract

This panel dataset provides an assessment of the independence of central banks in 21 OECD countries (excluding the Eurozone), focusing on their monetary policy autonomy as determined by legislation in 2010, 2015, and 2020. Our data collection adopts a novel approach, building upon the innovated methodology proposed by Cukierman et al. (1992), while incorporating revised components of the index that place greater emphasis on current standards of central bank independence. Additionally, we introduce new criteria to evaluate budgetary independence, an important aspect of central bank autonomy (Swinburne and Castello-Branco, 1991). The dataset serves as a valuable resource for empirical studies seeking to analyze the impact of monetary policy independence on economic performance. Furthermore, policymakers can draw insights from this index to enhance legislative frameworks and promote stronger performance in central bank independence.

Specifications Table

**Table:** 

Subject	Macroeconomics
Specific subject area	Central bank independence
Type of data	Table
How the data were acquired	To construct the panel dataset, we employed a novel central bank independence (CBI) index, which is introduced in this paper and builds upon the methodology developed by Cukierman et al. (1992). This index systematically breaks down independence into 14 distinct elements, categorized as personal, functional-institutional, financial, and budgetary independence, drawing inspiration from the insightful analysis of independence aspects discussed by Bini Smaghi (2008). Our data acquisition process involved detailed evaluating of the CBI for the years 2010, 2015, and 2020, aligning with the corresponding collection years of the IMF Central Bank Legislation Database. This meticulous approach enables researchers to utilize a robust panel dataset, providing a comprehensive perspective on the dynamics of CBI across the observed period.
Data format	Raw
Description of data collection	We collected the data by primarily reviewing the IMF Central Bank Legislation database and legislation from 21 OECD countries (excluding the Eurozone). The process of data normalization was employed and is thoroughly described in the accompanying paper.
Data source location	21 OECD countries (excluding the Eurozone): AUS, CAN, CHL, COL, CRI, CZE, DNK, HUN, ISL, ISR, JAP, KOR, MEX, NZL, NOR, POL, SWE, CHE, TUR, GBR, USA.
Data accessibility	Repository name: UK Data Service Data identification number: 10.5255/UKDA-SN-856672 Direct URL to data: https://reshare.ukdataservice.ac.uk/856672/

## 1 Value of the Data

• This unique dataset improves upon existing central bank independence indices, aligning better with current independence standards.
• It proves valuable for econometricians and lawmakers alike, facilitating analysis of the significance of central bank independence and its impact on economic performance.
• Furthermore, it enables comparisons of independence levels across different central banks.

## 2 Objective

We developed this dataset using a novel central bank independence index (presented in this paper) to provide an improved and comprehensive measure of central bank independence that aligns more with current standards. The latest datasets on central bank independence either follow the methodology of Cukierman et al. [9], see Garriga [12], which was designed for independence standards in the last century; or use too narrow index, which hardly captures the complexity of the CBI, see Arnone and Romelli [1].

## 3 Data Description

We collected the presented panel data using the methodology described in detail in the section below (refer to page 9) based on the collection years 2010, 2015, and 2020 from the IMF Central Bank Legislation database crosschecking it with the disposable official legislation of the analyzed central banks.

The first column of the dataset signifies the cross-sectional units, i.e., countries. The second column designates the temporal unit corresponding to each country identified in the first column. Subsequent columns, from the third to the sixth, encapsulate the points-level data on Personal, Functional and Institutional, Financial, and Budgetary independence of the central banks in each respective country.^1^

Upon examination of the mean values for the independence components (refer to Table 1), it becomes apparent that financial independence of the analyzed central banks is relatively low when juxtaposed with the other components. This disparity is, in part, attributable to the absence of crucial provisions in certain central banks, allowing them to lend to the government. Additionally, this component exhibits the highest sample variation. Conversely, provisions governing functional and institutional independence indicate a high emphasis on this component in the central bank legislation of the examined countries.

**Table 1:** Descriptive statistics of the central bank independence components.

Independence	N	Mean	St. Dev.	Min	Max
Personal	21	2.380	0.632	0.750	3.500
Functional and institutional	21	2.425	0.564	0.800	3.000
Financial	21	1.552	0.975	0	3.250
Budgetary	21	2.131	0.490	1	3

*Note:* This table presents the descriptive statistics of the central bank independence components, revealing a low performance of the analyzed central banks in financial independence.

The panel dataset reveals that the highest *de jure* personal independence is observed in Japan and Sweden. Conversely, Turkey in 2020, Norway, and Iceland in 2010 and 2015 exhibit considerably lower levels of personal independence. Exemplary legal provisions pertaining to functional and institutional independence, as per the index definition, are enacted in Chile. In contrast, the index suggests room for improvement in the functional and institutional independence of Korea and New Zealand. Chile demonstrates a robust financial independence, while Australia, Great Britain, New Zealand (primarily due to the absence of relevant provisions), Colombia, and Japan exhibit weaker financial independence. Costa Rica achieves a full-score in budgetary independence, whereas Japan fares relatively poorly in this regard.

Columns seven and eight of the dataset encompass the aggregated overall independence level, computed using Eq. (1) from the aforementioned subcomponents. The aggregate in column seven employs an equal subcomponents weights approach, while the measure in column eight utilizes judgment-based weights, as elaborated upon in the discussion below Table 4.^2^

Although the dataset is designed as panel data, there is generally little variation in the overall *de jure* independence between years, as the analyzed parts of legislation remained mostly unchanged. However, we observed a significant decrease in independence for the central bank of Turkey and an increase in independence for the central banks of Costa Rica and Iceland between 2015 and 2020. Additionally, we noticed less significant changes in independence for the central banks of Hungary, Norway, and Great Britain. The complete dataset is stored in a public repository.

**Fig. 1:**
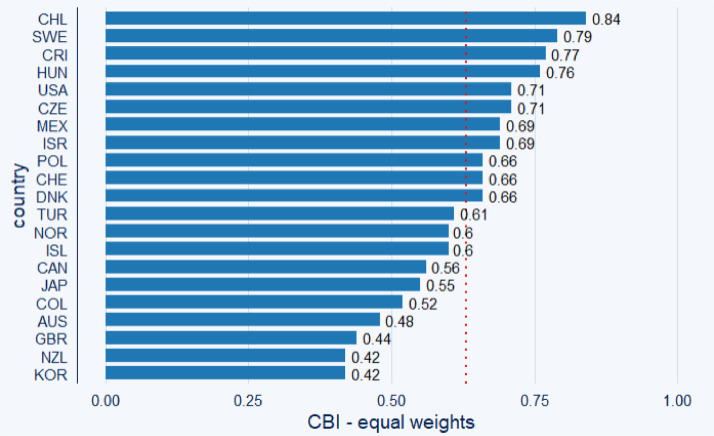
Central bank independence (CBI) of the OECD 21 countries (excluding Eurozone) computed using equal weights. *Note:* Using the equal weights approach, the sample mean of central bank independence is 0.626 (red dotted line). Chile had the highest average *de jure* independence level over 2010, 2015, and 2020. Data source: Own calculations.

**Fig. 2:**
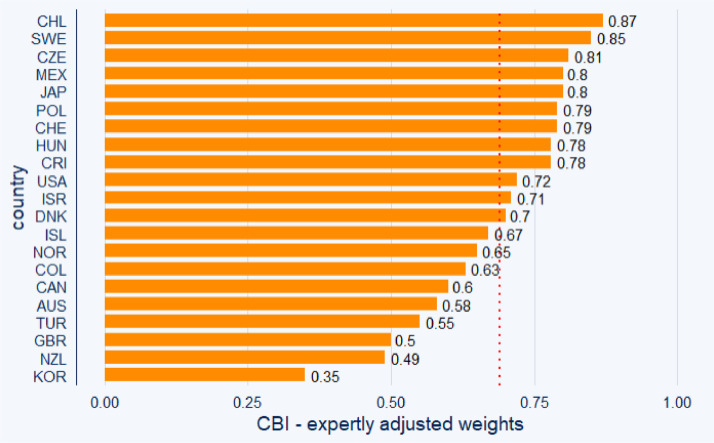
Central bank independence (CBI) of the OECD 21 countries (excluding Eurozone) computed using expertly adjusted weights. *Note:* Using the expertly adjusted weights approach, the sample mean of central bank independence is 0.687 (red dotted line). Chile had the highest average *de jure* independence level over 2010, 2015, and 2020. Data source: Own calculations.

To efficiently visualize and describe our data, considering the limited time variability of the index, we calculated the mean value of the index for the collection years 2010, 2015, and 2020. Figs. 1 and 2 display bar charts depicting the mean value of central bank independence for the analyzed periods. Fig. 1 presents the index using equal weights for all categories, while Fig. 2 presents the index with expertly adjusted weights (for a discussion on the weight selection problem, please refer to page 12 and Table 4).

Both Figs. 1 and 2 indicate that the central banks of Chile and Sweden have the highest *de jure* independence among the analyzed central banks. In contrast, our index suggests relatively lower independence levels for the central banks of Korea and New Zealand.

Note that the selection of the weights matters, as the rank in the independence comparison changes for some of the central banks between Figs. 1 and 2 (take USA as an example).

**Fig. 3:**
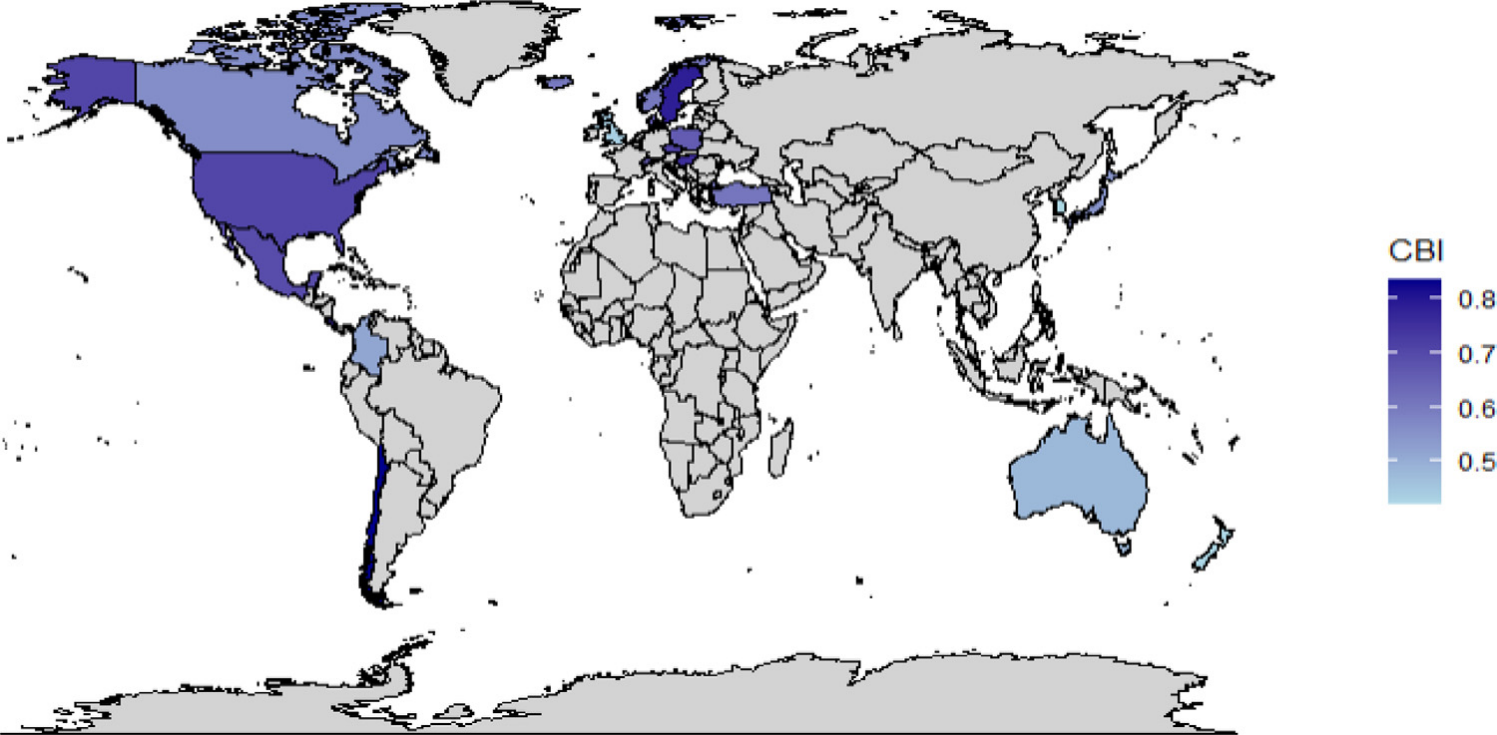
Map of central bank independence (CBI) of the OECD 21 countries (excluding Eurozone) computed using equal weights. *Note:* Using the equal weights approach, Chile, Sweden and Costa Rica have high central bank independence levels. Data source: Own calculations.

**Fig. 4:**
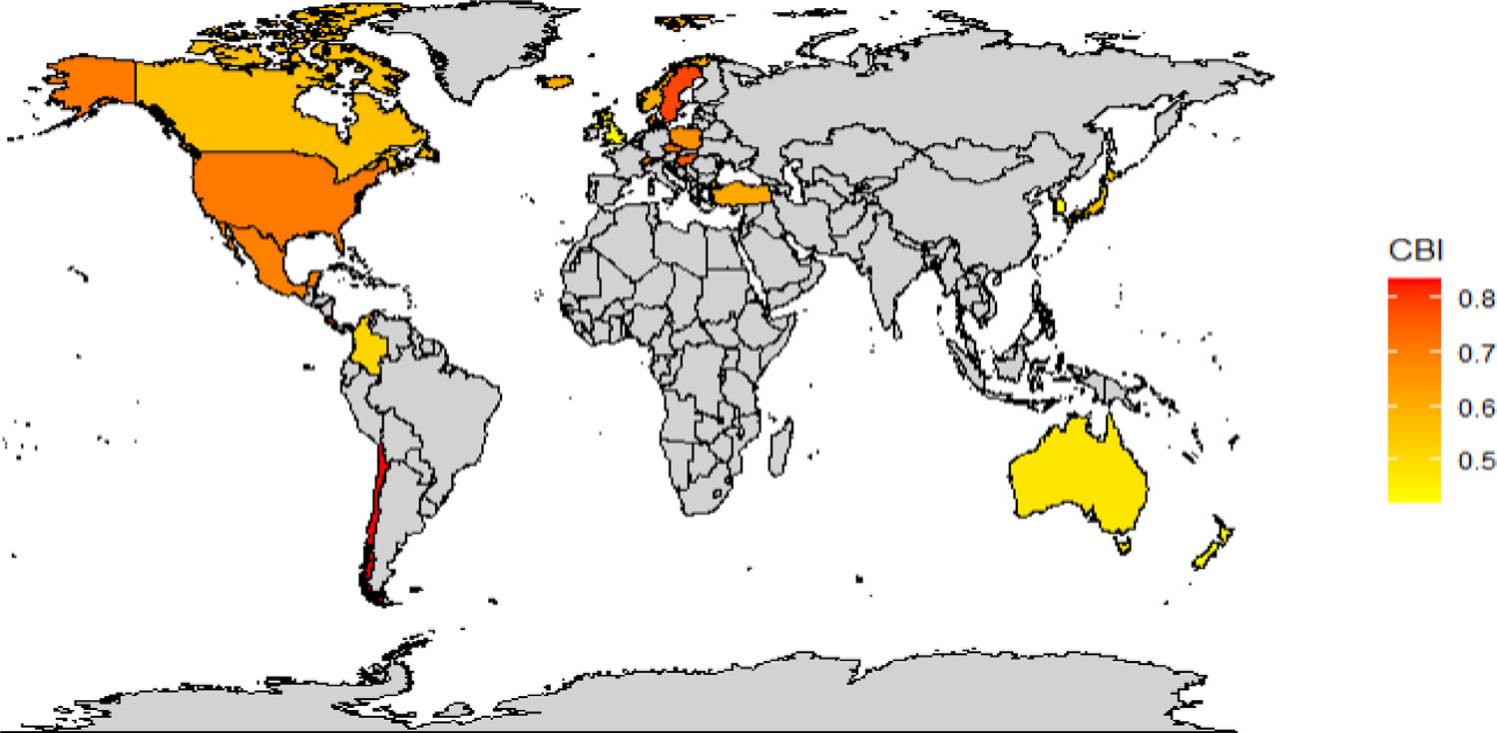
Map of central bank independence (CBI) of the OECD 21 countries (excluding Eurozone) computed using expertly adjusted weights. *Note:* Using the expertly adjusted weights approach, Chile, Sweden and the Czech Republic have high central bank independence level. Data source: Own calculations.

When we plot the data on a world map, we can observe that the examined countries whose central banks are members of the Eurosystem have relatively high independence levels regardless of the index-weights selection (see Fig. 3, plotting the index with equal weights; and Fig. 4, plotting the index with expertly adjusted weights). In contrast, the central banks of the examined Commonwealth countries exhibit relatively lower independence.

The information provided graphically by Fig. 1, Fig. 2, Fig. 3, Fig. 4 is also presented in Table 2, offering descriptive statistics of the panel dataset.

**Table 2:** Descriptive statistics of the panel dataset.

	*N*	Mean	St. Dev.	Min	Max
Equal weights	21	0.626	0.123	0.420	0.840
Judgmented weights	21	0.687	0.136	0.350	0.870

*Note:* In advance to Fig. 1, Fig. 2, Fig. 3, Fig. 4, we can observe a relatively high standard deviation in central bank independence among the examined countries.

## 4 Experimental Design, Materials and Methods

The methodology behind the dataset, which is visualized in the previous sections in Fig. 1, Fig. 2, Fig. 3, Fig. 4, is based on Cukierman [8] and Cukierman, Webb, and Neyapti (CWN) [9], which have been widely used in empirical studies on central bank independence [2,17]. However, critiques of the CWN approach highlight its omission of important independence components such as budgetary independence and bank board autonomy [7,11].

To address these critiques and overcome theoretical limitations, we propose a similar index that addresses these concerns and simplifies computationally intensive aspects of the CWN approach. The index components are primarily derived from the central banks' charters of OECD countries over the last decade, maximizing objectivity and minimizing subjective evaluations. Additionally, we verified that our component selection aligns with the theoretical framework outlined by Swinburne et al. [16]. We define four main components of central bank independence following Bini Smaghi [5]: personal, functional & institutional, financial, and budgetary independence, further decomposed into 14 criteria in total, each coded on a scale ranging from 0 (lowest independence level) to 1 (highest independence level).

Regarding personal independence, we focus on the mandate of the monetary policy decision-making body (bank board) and evaluate the personal independence of individual board members separately, weighing their decision power. This approach is more precise compared to CWN, which assesses personal independence based solely on the chairman. Notably, the current central banks' legislation standards often grant the chairman the advantage of resolving tie votes rather than sole decision-making authority, and regular board members may have different mandate terms. Omitting these standards would significantly affect the resulting index values.

The index favors the legislature, ensuring that bank board members have longer office terms than the subjects who appointed them. It emphasizes that appointments should not be monocratically performed and dependent on the executive. Additionally, the index rewards legislation that prevents easy removal of bank board members based on their decisions and eliminates the cumulation of bank board membership and office in government.

For functional & institutional independence, we examine whether the central bank is sufficiently separated from legislative and executive powers when deciding on its monetary policy target and main policy instruments. Therefore, the high values in this part of the index are awarded if the central bank: decides on monetary policy without the inference of other public offices; has priority in case a conflict with another public body arises; states its policy objective alone. This approach diverges to CWN as we believe that price stability as a predetermined goal is not a manifestation of higher independence than the state in which the bank sets its targets itself. In the financial independence component, we study the potential of subjects to affect monetary policy through central bank's financial support. To make the index computationally efficient, we simplify the CWN criteria significantly. Unlike CWN, we do not believe that financial independence is a more important component than personal and functional & institutional independence (see the arguments below 4) and hence it does not require such a comprehensive analysis. Moreover, many of the CWN criteria do not align with current legislation standards, which reduces comparability among banks with and without comprehensive financial independence amendments. Thus, we propose four major criteria that cover the majority of financial independence aspects. We assume that restrictions on non-securitized lending, direct securitized lending and indirect securitized lending via purchases with private institutions put less political pressures on the central bankers and lead to higher independence level. Similarly, we find the restrictions to lending to private institutions beyond standard open market operations to be beneficial to financial independence.

The final component of the index is budgetary independence, which evaluates potential influences on the bank board's monetary policy decisions through control over the central bank's access to budgetary resources. By including this component, we deviate from CWN, which completely omits budgetary independence. The index favors legislation that separates the central bank's budget from other institutions, ensures that the budget is determined without interference from other public bodies, and does not require approval of financial statements from bodies lacking independence.

The detailed structure of the index, including the groups and criteria, is described in Table 3.

**Table 3:** The detailed structure of the index.

Independence group	Criteria	Legal characteristics	Score
1. Personal	a) Office term	Longer than appointer‘s	1
		Same as appointer‘s	1/2
		Shorter than appointer‘s	0
	b) Appointer*	Bank board	1
		At least two bodies none of them holding executive power	3/4
		At least two bodies none of one of them holding executive power	2/4
		Single body which does not hold executive power	1/4
		Single body which holds executive power	0
	c) Removal	Impossible	1
		For reasons unrelated to monetary policy and the law allows to defend against removal act at court	7/8
		For reasons unrelated to monetary policy and the law does not allow to defend against removal act at court	6/8
		At discretion of bank board	5/8
		At discretion of joint decision of at least two non-executive bodies none of them holding executive power	4/8
		At discretion of joint decision of at least two bodies one of them holding executive power	3/8
		At discretion of single body which does not hold executive power	2/8
		At discretion of single body which holds executive power	1/8
	d) Disqualification	Office in government fully disqualifies	1
		Office in government disqualifies unless non-executive body authorizes	2/3
		Office in government disqualifies unless executive body authorizes	1/3
		Incompatibility is not enacted	0
2. Functional and institutional	a) Monetary policy decision-making	Bank board	1
		Bank board jointly with body holding executive power	2/3
		Bank board has only advisory role	1/3
		Bank board has no power	0
	b) Final word in case of conflict	Bank board, or the confict cannot happen	1
		Bank board in case of monetary policy decisions, another body in case of other decisions	4/5
		Bank board after consultations with other bodies	3/5
		Non-executive body	2/5
		Executive body but the priority is conditional to some restriction or process	1/5
		Executive body has unconditional priority	0
	c) Policy objective	Formulated by the bank	1
		Formulated jointly by the bank and non-executive body or bodies⁎⁎	4/5
		Formulated jointly by the bank and other bodies one of them holding executive power⁎⁎	3/5
		Formulated jointly by executive and non-executive body (bank has no say)	2/5
		Formulated solely by non-executive body	1/5
		Formulated solely by executive body	0
3. Financial	a) Non-securitized lending to Government (advances)	Prohibited	1
		Allowed under state of emergency and with restrictions	3/4
		Allowed with restrictions	2/4
		Allowed only under state of emergency	1/4
		Allowed unlimitedly or not regulated	0
	b) Direct securitized lending to Government	Prohibited	1
		Allowed under state of emergency and with restrictions	3/4
		Allowed with restrictions	2/4
		Allowed only under state of emergency	1/4
		Allowed unlimitedly or not regulated	0
	c) Indirect securitized lending to Government	Prohibited	1
		Allowed under state of emergency and with restrictions	3/4
		Allowed with restrictions	2/4
		Allowed only under state of emergency	1/4
		Allowed unlimitedly or not regulated	0
	d) Lending to private sector (except for standard free market operations)	Prohibited	1
		Allowed under state of emergency and with restrictions	3/4
		Allowed with restrictions	2/4
		Allowed only under state of emergency	1/4
		Allowed unlimitedly or not regulated	0
4. Budgetary independence	a) Independent budget	Completely	1
		Own budget but profit/deficit is transferred/covered by state budget	1/2
		Bank's finances belong under state budget	0
	b) Decision about budget	Bank solely or not enacted***	1
		Joint decision of bank and at least one non-executive body	3/4
		Joint decision of bank and at least one executive body	2/4
		Non-executive body solely	1/4
		Executive body solely	0
	c) Financial report revision	Revision is not enacted or is performed by bank's independent body or independent external office	1
		Subject to approval of non-executive body	1/2
		Subject to approval of executive body	0

*Note:* This table shows the detailed decomposition of the novel central bank independence index.

⁎ In cases where the chairman or board member is elected to the office by an independent entity, such as bank managers, we consider such appointments to be harmless to independence. Therefore, such cases are evaluated as 1, indicating the highest level of independence in the *Appointer*’s criterion.

⁎⁎ We even consider the general legal specification of the central bank's objective, such as price stability, as potentially encroaching on the central bank's autonomy. This is because when the legislature explicitly dictates the bank's objective, it can diminish the central bank's independence.

Our index is computed from four independence groups, each with a defined maximum number of points a central bank can obtain. For personal independence and financial independence, the maximum is four points per category, while for functional & institutional and budgetary independence, the maximum is three points per category. The maximum total points for each central bank is 14. Each group is weighted based on its importance for overall independence, with a total weight sum of 1. The index (*CBI*) is formally described by the following formula:

(1) $$CBI=\;\frac{Pers_{act}}{Pers_{max}}\times w_{pers}+\frac{Fun_{act}}{Fun_{max}}\times w_{fun}+\frac{Fin_{act}}{Fin_{max}}\times w_{fin}+\frac{Bud_{act}}{Bud_{max}}\times w_{bud}$$

where variables with subscript *act* represent actual values obtained by the central bank in a given independence category, variables with subscript *max* represent maximum values that can be obtained in each category, and w denotes weight for the respective category. This normalization ensures the index remains within the interval of 0 and 1.

Determining appropriate weights for the index poses a significant challenge. In the literature, two main approaches exist, both with their limitations. The first option is to assign equal weights to all criteria and groups while adjusting for the number of criteria in each category [13]. This approach reduces subjective judgments but may introduce bias when certain independence components are more important for overall independence. Contrary to Jasmine et al. [13], we opt for a simpler computation in formula (1) and do not utilize a two-step weights and normalization procedure. This approach addresses the issue of implicit weight increase with a higher number of criteria in a given category. Assuming the equal weights approach, each of the independence categories is weighted by 0.25.

The second approach found in the literature relies on expert judgments to determine weights [[8], [9]]. This allows for better reflecting the higher importance of certain categories but introduces subjectivity bias.

As we do not consider one approach superior to the other, we provide and publish data obtained using both methods with a slight modification of the equal weights approach shown in Eq. (1). We encourage researchers to use our published data on each independence category and develop more robust approaches to determine the importance of the categories.

Our expert weights judgment is described in Table 4.

**Table 4:** Expert selection of weights.

Independence category	Personal	Functional & institutional	Financial	Budgetary
Weight	0.35	0.5	0.1	0.05

*Note:* Following the arguments mentioned below, we recommend to select the weights as shown in this table.

The main reason why central bank independence has been promoted in the last decades [[4], [6], [10], [14]] is that it ensures that the interests of the central bank described by its goals are not jeopardized by conflictual political interests of another public bodies. Therefore, we consider the functional & institutional independence to be critical for the overall independence level. When this independence group is violated and the bank is subordinated to the will of another public body, the importance of the remaining independence groups diminishes.^3^

The personal independence shall be also granted high importance, because its low level increases the problems connected with bad performance in financial and budgetary independence. Assume a central bank whose bank board can be easily replaced by executive. If this central bank has unlimited option for government lending, then the executive can appoint board members that will help it to finance government debt. In contrast, if the central bank has an independent bank board that has no incentive to act in a way that favors executive, the problem with low performance in financial independence is less severe, as the bank board will lend to government only to the best of their knowledge and conscience.

Financial independence has shown to be important with respect to hyperinflation cases in some countries (see Beckerman, [3]; Kavila and Le Roux, [15]). Nevertheless, as noted above, it is redundant in case, that the central bank is sufficiently functionally & institutionally and personally independent. Therefore, we assign it a much lower value compared to CWN.

Lastly, we acknowledge the potential influence of the authority deciding on the central bank's expenditures on the bank board. However, we deem this channel relatively negligible compared to other independence groups. Consequently, we suggest that budgetary independence should carry the lowest weight in the aggregated index.

## CRediT Author Statement

**Tomáš Pokorný:** Conceptualization, Methodology, Index computation, Investigation, Data Curation, Writing - Original Draft, Writing – Review & Editing, Visualization - graphical, Project administration, Funding acquisition. **Adam Ruschka:** Index computation, Data Curation, Visualization – tables, Writing – Review & Editing. **Helena Chytilová:** Resources, Writing - Review & Editing, Supervision

## Data Availability

Central Bank Independence in 21 OECD Countries: Panel Dataset, 2010-2020 (Original data) (UK Data Service).

## Appendix Supplementary materials

Image, application 1

## Footnotes

1 The dataset is available in the public repositary.

2 The dataset is available in the public repositary.

3 Note that the weights are adjusted solely by our expert judgements based on the presented arguments and our experience in central banking.
