# Supplementary material for: Panel dataset on de jure central bank independence in 21 OECD countries (excluding the Eurozone)
Source: Data Brief. 2024 Feb 1;53:110094. doi: 10.1016/j.dib.2024.110094 (PMC10859258; doi:10.1016/j.dib.2024.110094)
Supplement: Supplementary file 1 [file mmc1.docx]

**Ethical Statement**

This research manuscript adheres to the ethical standards and guidelines set forth by Elsevier and the Data in Brief. The authors declare that:

1. **Authorship and Contributions:**
   - All listed authors have made significant contributions to the research and writing of this manuscript.
   - All authors are aware of and agree to the submission of this manuscript.
2. **Originality and Plagiarism:**
   - The manuscript is an original work, and the authors have appropriately cited and credited the contributions of others.
   - No part of this manuscript has been plagiarized, and all sources have been duly acknowledged.
3. **Data Integrity and Transparency:**
   - Data presented in the manuscript are accurate and have been collected, analysed, and interpreted with integrity.
   - Any data manipulation or image enhancement has been clearly disclosed.
4. **Informed Consent:**
   - No human and/or animal subjects were involved.
5. **Conflict of Interest:**
   - The authors disclose any financial or personal relationships that could be perceived as influencing the research or its interpretation.
   - Any potential conflicts of interest have been appropriately addressed.
6. **Review and Approval:**
   - The manuscript has been reviewed and approved by all authors before submission.
7. **Compliance with Ethical Standards:**
   - The research has been conducted in compliance with all relevant ethical standards, including but not limited to those outlined in the Data in Brief guidelines.
8. **Funding:**
   - The sources of funding for the research have been disclosed, and there is no conflict of interest associated with the funding.

The authors understand that any breach of the above-mentioned ethical standards may result in appropriate action by the editorial board of Data in Brief.
